# Regulation of lipid metabolism-related gene expression in whole blood cells of normo- and dyslipidemic men after fish oil supplementation

**DOI:** 10.1186/1476-511X-11-172

**Published:** 2012-12-14

**Authors:** Simone Schmidt, Janina Willers, Frank Stahl, Kai-Oliver Mutz, Thomas Scheper, Andreas Hahn, Jan Philipp Schuchardt

**Affiliations:** 1 Institute of Food Science and Human Nutrition, Faculty of Natural Sciences at the Leibniz University of Hannover, Am Kleinen Felde 30, Hannover, 30167, Germany; 2 Institute of Technical Chemistry, Faculty of Natural Sciences at the Leibniz University of Hannover, Callinstr. 5, Hannover, 30167, Germany

**Keywords:** Omega-3 fatty acids, TG lowering, Dyslipidemia, PPARα, HNF, RXR

## Abstract

**Background:**

Beneficial effects of omega-3 polyunsaturated fatty acids (n-3 PUFAs) on the lipid levels of dyslipidemic subjects are widely described in the literature. However, the underlying molecular mechanisms are largely unknown. The aim of this study was to investigate the effects of n-3 PUFAs on the expression of lipid metabolism-related genes in normo- and dyslipidemic men to unveil potential genes and pathways affecting lipid metabolism.

**Methods:**

Ten normo- and ten dyslipidemic men were supplemented for twelve weeks with six fish oil capsules per day, providing 1.14 g docosahexaenoic acid and 1.56 g eicosapentaenoic acid. The gene expression levels were determined by whole genome microarray analysis and quantitative real-time polymerase chain reaction.

**Results:**

Several transcription factors (peroxisome proliferator-activated receptor α (PPARα), retinoid X receptor (RXR) α, RXRγ, hepatic nuclear factor (HNF) 6, and HNF1ß) as well as other genes related to triacylglycerol (TG) synthesis or high-density lipoprotein (HDL-C) and cholesterol metabolism (phospholipids transfer protein, ATP-binding cassette sub-family G member 5, 2-acylglycerol O-acyltransferase (MOGAT) 3, MOGAT2, diacylglycerol O-acyltransferase 1, sterol O-acyltransferase 1, apolipoprotein CII, and low-density lipoprotein receptor) were regulated after n-3 PUFA supplementation, especially in dyslipidemic men.

**Conclusion:**

Gene expression analyses revealed several possible molecular pathways by which n-3 PUFAs lower the TG level and increase the HDL-C and low-density lipoprotein level, whereupon the regulation of PPARα appear to play a central role.

**Trial registration:**

ClinicalTrials.gov (ID: NCT01089231)

## Background

Fish oil (FO) and its principal omega-3 polyunsaturated fatty acids (n-3 PUFAs), eicosapentaenoic acid (EPA, 20:5n-3) and docosahexaenoic acid (DHA, 22:6n-3), have shown beneficial effects on the lipid profile in numerous interventional studies [1-3]. Primarily, n-3 PUFAs lower triacylglycerol (TG) levels, especially in subjects with hypertriglyceridemia [4-6]. The TG-lowering effect of n-3 PUFAs is more pronounced at higher baseline TG levels [3] and appears to be dose-dependent [1,3]. The recommended daily intake of n-3 PUFAs for TG lowering in hypertriglyceridemic subjects ranges from 2 to 5 g/d; amounts which could only be reached by supplementation [7,8]. However, moderate n-3 PUFA doses (1.68 g/d) are similarly efficient at reducing elevated TG levels in subjects with mild hypertriglyceridemia [9].

Numerous mechanisms have been proposed as contributors to the TG-lowering effect of n-3 PUFAs, for example, by reducing very low-density lipoprotein- (VLDL) TG synthesis and secretion from the liver, or by enhancing the TG clearance of circulating VLDL and chylomicron particles [10]. Beyond TG lowering, n-3 PUFAs additionally affect the high-density lipoprotein cholesterol (HDL-C) metabolism by elevating the cholesterol-rich HDL2 subtype and reducing the TG-rich HDL3 subtype [11,12]. Beside these beneficial effects, studies have repeatedly shown that n-3 PUFAs increase low-density lipoprotein cholesterol (LDL-C) levels, which may result from the conversion of VLDL to LDL-C [13].

The molecular mechanisms by which n-3 PUFAs modify the lipid metabolism are not completely clarified. The regulation of gene expression is believed to be a key mechanism of how n-3 PUFAs mediate their functions. Specifically, n-3 PUFAs can modulate the activity of several transcription factors, such as sterol regulatory element-binding protein (SREBP) 1 [14], hepatic nuclear factor (HNF) 4α [15], liver X receptors [16], retinoid X receptor (RXR) [17], farnesoid X receptor [18], and peroxisome proliferator-activated receptors (PPARs) [19], resulting in an altered expression of corresponding target genes [20-24]. Although it is known that these genes, or rather their products, play eminent roles in the regulation of the lipid metabolism, the influence of n-3 PUFAs on a number of additional lipid metabolism-related genes and involved pathways remain to be discovered. Unravelling these connections may contribute to the understanding of the molecular mechanisms explaining the physiological functions of n-3 PUFAs.

The approach of this interventional trial was to monitor gene expression changes in normo- and dyslipidemic male subjects after n-3 PUFA supplementation using whole blood samples. With a focus on lipid metabolism-related genes, we aimed to not only identify genes and associated pathways that confirm already known mechanisms, but also to point out alternative mechanisms of how n-3 PUFAs affect lipid metabolism.

## Methods

This controlled, parallel group intervention study was conducted at the Institute of Food Science and Human Nutrition, Leibniz University of Hannover, Germany, and performed with respect to GCP (Good Clinical Practice) Guidelines. The approval of the Freiburg Ethics Commission International (FECI) was received. The clinical investigation was registered at ClinicalTrials.gov with the identification number NCT01089231.

Parts of this study have been published recently. In two other publications, we presented regulated pathways, which have been discovered in normo- and dyslipidemic male subjects after FO supplementation [25] as well as regulated antioxidative genes expression [26]. Therefore, selection criteria for the study subjects, the study design as well as the sample collection for gene expression analyses and methodical procedure of microarray experiments are described elsewhere [26].

### Determination of fasting serum lipids and apolipoprotein B48 concentration

Fasting venous blood samples were collected into BD Vacutainer® Blood Collection Tubes (Becton Dickinson, Heidelberg, Germany) at baseline (t_0_) and after twelve weeks (t_12_) of supplementation. The plasma lipid levels were determined by specific enzymatic colour reactions from an external contract laboratory (LADR, Hannover, Germany).

Apolipoprotein (Apo) B48 concentration in fasting serum at baseline and after twelve weeks was determined in 12 subjects using the Shibayagi human Apo B-48 enzyme linked immunosorbent assay (ELISA) kit (Xceltis GmbH, Mannheim, Germany). Analysis was performed in accordance to the manufacturer’s recommended procedures.

### Quantitative real-time polymerase chain reaction (qRT-PCR) and data analysis

In order to quantify the expression levels of selected genes, equal amounts of cDNA were synthesized using 2.0 μg of purified RNA and M-MLV reverse transcriptase (Promega, Mannheim, Germany), as well as random hexamer (Fermentas, St. Leon-Rot, Germany) and oligo(dT) primers (Carl Roth, Karlsruhe, Germany). Synthesized cDNA was diluted 1:20 with nuclease-free water and used for the qRT-PCR together with iQ SYBR Green Supermix (Bio-Rad Laboratories, Hercules, Ca, USA) and 5 pmol of both forward and reverse primers. The sequences for target and reference genes were retrieved from GenBank and the primers applied were manually designed with the Primer-BLAST tool of the National Centre for Biotechnology Information, which is based on the program Primer3 [27]. The primer sequences used are listed in Table 1. Glyceraldehyde-3-phosphate dehydrogenase (GAPDH) and ribosomal protein S2 (RPS2) were identified as the most stable reference genes by the freely available algorithm geNorm version 3.5.

**Table 1:** Nucleotide sequences of primers for quantitative real-time polymerase chain reaction

	**Gene symbol**	**RefSeq_ID**	**Sequences (5’- > 3’)**	
**Target genes**	Apo C II	NM_000483.3	forward	GCTCCCCCTTCCCAGTAGCTCT
			reverse	TTCACTGCTTTATTCCCATGGACCC
	LDLR	NM_000527.3	forward	GGGGCCCTGTGTAGGGGGTT
			reverse	AAAGTGACACCCATCTCCCAGAAGC
**Reference genes**	GAPDH	NM_002046.3	forward	AAGGTGGTGAAGCAGGCGTCG
			reverse	AATGCCAGCCCCAGCGTCAAAG
	RPS2	NM_002952.3	forward	GCAACTTCGCCAAGGCCACCTT
			reverse	TGGGTCTTGACGAGGTGGTCAGT

### Statistics

Statistical analysis of blood lipids was processed with SPSS software version 17.0 (SPSS Inc., Chicago, IL, USA). Statistical analyses were based on per protocol population, defined as subjects completing all visits not infringing the study protocol. The results are presented as mean ± SD (Table 2). Statistical analysis of Apo B48 levels was performed with the statistical package R version 2.15.0. Differences between t_0_ and t_12_ were tested within groups by paired *t*-test and differences between groups were examined by *t*-test. P-values ≤0.05 were interpreted as statistically significant.

**Table 2:** **Serum lipid levels of the normo- and dyslipidemic men at baseline (t**_**0**_**) and after supplementation with fish oil over twelve weeks (t**_**12**_**)**

**Parameters**	**Normolipidemic (n = 9)**		**Dyslipidemic (n = 7)**	
	**t**_**0**_	**t**_**12**_	**t**_**0**_	**t**_**12**_
Total cholesterol [mg/dl]	183.33 ± 13.88 ^a^	190.56 ± 21.88 ^b^	272.86 ± 67.17 ^*a*^	278.40 ± 45.21 ^b^
Triacylglycerol [mg/dl]	82.22 ± 37.42 ^a^	63.00 ± 14.09 ^b^	362.00 ± 284.62^*a*^	262.14 ± 153.52 ^b^
High-density lipoprotein [mg/dl]	58.67 ± 10.92	65.67 ± 15.23 ^c_T^	45.86 ± 6.15	52.14 ± 9.84 ^c^
Low-density lipoprotein [mg/dl]	108.33 ± 13.54	112.33 ± 16.88 ^b^	146.60 ± 6.43	176.20 ± 20.56 ^b^^c^
LDL-C/HDL-C quotient	1.90 ± 0.37 ^*a*^	1.80 ± 0.51 ^b^	3.10 ± 0.47 ^*a*^	3.28 ± 0.89 ^b^

a: p < 0.05 (Changes of means at baseline were evaluated between normolipidemic and dyslipidemic subjects by Student’s *t*-test).

b: p < 0.05 (Changes of means after twelve weeks of supplementation were evaluated between normolipidemic and dyslipidemic subjects by Student’s *t*-test).

c: p < 0.05 (Changes between t_0_ and t_12_ were evaluated within groups by Student’s *t*-test for dependent samples).

_T: p < 0.1 (trend of significance).

Statistical analysis of the expression ratios of genes, which were quantified by qRT-PCR, were calculated with the Gene Expression Macro tool (Bio-Rad), which is based on the algorithm of geNorm [28]. Firstly, normalization factors were calculated from the geometric mean of the reference genes GAPDH and RPS2. Furthermore, the baseline values of the normolipidemic group were defined as control values so that relative expression values could be calculated. Therefore, the baseline samples of the normolipidemic group are given a value of 1.

## Results

### Subject characteristics

All subjects completed the study. One normo- and three dyslipidemic subjects had to be excluded from the analyses because of low RNA yield (n = 3) and consumption of medication that led to exclusion (n = 1). Thus, data were available from nine normolipidemic and seven dyslipidemic subjects for each investigation time point.

Age and mean weight at baseline did not show any differences between either group. However, the dyslipidemic subjects had a significantly higher BMI than the normolipidemic subjects (28.13 kg/m^2^ vs. 23.66 kg/m^2^, respectively). Subjects of the dyslipidemic group can be characterised as pre-obese (BMI 25–30), which is, among others, an underlying cause for dyslipidemia. Nevertheless, the BMI was not changed by dietary intervention in either of the study groups.

### Changes of blood lipids and apolipoprotein B48 levels

According to the inclusion criteria, dyslipidemic subjects had significantly higher TC and TG levels as well as a higher LDL-C/HDL-C ratio at baseline (Table 2). After twelve weeks of supplementation with FO, the group differences in TC and TG levels and the LDL-C/HDL-C ratio remained unaffected and similar to differences observed at baseline. TG levels decreased both in normolipidemic (−19.2 mg/dl; -23.34%) and dyslipidemic subjects (−99.86 mg/dl, -27.59%). The difference between both groups was not statistically significant. The LDL-C level increased significantly after FO supplementation in dyslipidemic subjects (29.6 mg/dl; 20.19%), whereas the effect in normolipidemic subjects was marginal (4.0 mg/dl; 3.69%). Furthermore, FO supplementation resulted in a significant increase in the HDL-C levels in dyslipidemic subjects (6.28 mg/dl; 13.69%), whereas the HDL-C increase in normolipidemic subjects (7.0 mg/dl; 11.93%) showed a tendency towards statistical significance.

**Figure 1:**
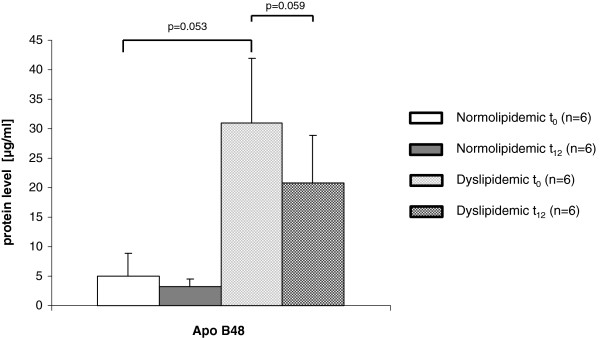
**Protein levels of apolipoprotein B48 in normolipidemic and dyslipidemic men.** Serum protein levels of apolipoprotein B48 (Apo B48) was determined by ELISA in normo- and dyslipidemic men before (t_0_) and after twelve weeks (t_12_) of fish oil supplementation. Differences between t_0_ and t_12_ protein levels were tested by a paired *t*-test, and differences between groups at each time point were tested by unpaired *t*-test using the statistical package R version 2.15.0.

The protein level of Apo B48 in dyslipidemic subjects at baseline was six times higher compared to normolipidemic subjects (p = 0.053) (Figure 1). After twelve weeks of FO supplementation, Apo B48 levels decreased both in normolipidemic (−1.8 μg/ml; -36%) and dyslipidemic subjects (−32.6 μg/ml; -33%), but only the decrease of the dyslipidemic subjects showed a tendency of significance (p = 0.059).

### Gene expression changes of lipid metabolism-related genes after n-3 PUFA supplementation

Microarray experiments revealed a transcriptional regulation of several transcription factors after FO supplementation, including PPAR alpha (PPARα), RXR alpha (RXRα), RXR gamma (RXRγ), HNF6, and HNF1ß (Table 3). While some transcription factors were similarly regulated in normolipidemic men, the expression was distinctly more strongly regulated in dyslipidemic men. Additionally, several genes related to TG synthesis and HDL-C and cholesterol metabolism were regulated in dyslipidemic men (Table 3). More precisely, the PPAR target gene phospholipid transfer protein (PLTP), as well as the ATP-binding cassette sub-family G member 5 (ABCG5) were up-regulated, while 2-acylglycerol O-acyltransferase 3 (MOGAT3), MOGAT2, diacylglycerol O-acyltransferase 1 (DGAT1), and sterol O-acyltransferase 1 (SOAT1) were down-regulated after FO supplementation in dyslipidemic men.

**Table 3:** Expression ratios of lipid metabolism-related genes

**Gene**	**Gene symbol**	**Entrez_ID**	**RefSeq_ID**	**dyslipidemic**			**normolipidemic**			**Function**
				**Ratio**	**Ratio**	**Ratio**	**Ratio**	**Ratio**	**Ratio**	
				**t**_**4h**_**:t**_**0**_	**t**_**1**_**:t**_**0**_	**t**_**12**_**:t**_**0**_	**t**_**4h**_**:t**_**0**_	**t**_**1**_**:t**_**0**_	**t**_**12**_**:t**_**0**_	
**Transcription factors**										
Peroxisome proliferator-activated receptor alpha	PPARA	5465	NM_001001928.2	−8.19^*^	2.72^**^	-	-	-	-	Regulation of the lipid metabolism
			NM_005036.4							
Retinoic X receptor RXR-alpha	RXRA	6256	NM_002957.4	-	−4.50^*^	-	3.14^*^	3.79^*^		
Retinoic acid receptor RXR-gamma	RXRG	6258	NM_006917.3	3.16^***^	4.79^***^	4.11^***^	−3.19^**^	-	−2.03^**^	
Hepatocyte nuclear factor 6	HNF6	3175	NM_004498.1	−2.27^*^	−4.89^*^	−3.43^*^	-	-	-	
Hepatocyte nuclear factor 1-beta	HNF1B	6928	NM_000458.2	-	−3.64^*^	−2.56^*^	-	-	-	
**Triacylglycerol synthesis**										
2-acylglycerol O-acyltransferase 3	MOGAT3	346606	NM_178176.2	-	−27.07^*^	−3.48^*^	-	-	-	TG synthesis
2-acylglycerol O-acyltransferase 2	MOGAT2	80168	NM_025098.2	−3.08^*^	-	-	-	-	-	
Diacylglycerol O-acyltransferase 1	DGAT1	8694	NM_012079.4	-	−2.67^*^	-	-	-	-	
**HDL-C metabolism**										
Phospholipid transfer protein	PLTP	5360	NM_006227.2	-	3.30^*^	4.15^*^	-	-	-	Modify HDL-C particles size
			NM_182676.1							
**Cholesterol metabolism**										
ATP-binding cassette sub-family G member 5	ABCG5	64240	NM_022436.2	-	4.06^*^	5.34^*^	-	-	-	Cholesterol efflux
sterol O-acyltransferase 1	SOAT1	6646	NM_003101.4	-	−2.45^*^	−2.37^**^	-	-	-	Cholesterol synthesis

Expression ratios were displayed for genes which were differentially expressed after four hours (t_4h_), one week (t_1_) and twelve weeks (t_12_) of fish oil supplementation in normolipidemic and dyslipidemic men.

-: no regulation.

^*^: p = 0.05.

^**^: p < 0.05.

^***^: p < 0.01.

**Figure 2:**
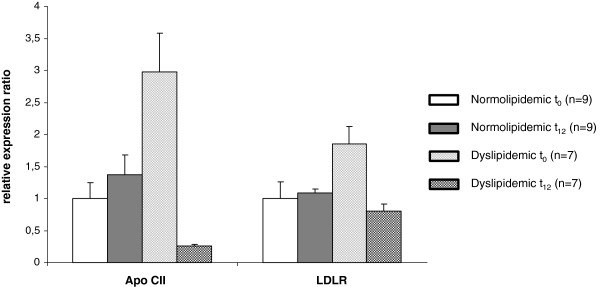
**Regulatory effects of eicosapentaenoic acid and docosahexaenoic acid on lipid metabolism related genes.** The Figure presented is based on the analysis of gene expression changes after fish oil supplementation in dyslipidemic male subjects. While bold text and black and grey arrows present findings discovered in this thesis, grey arrows symbolize that until now it has not been clarified if genes are target genes of PPARα. In addition, broken arrows symbolize further possible mechanisms of action based on findings from the literature.

Gene expression changes of Apo CII and LDL receptor (LDLR) were quantified by qRT-PCR (Figure 2). The expression of Apo CII in dyslipidemic subjects at baseline was three times higher compared to normolipidemic subjects (p = 0.05). After twelve weeks of FO supplementation, the expression of Apo CII was slightly up-regulated in normolipidemic subjects, and significantly down-regulated in dyslipidemic subjects (−57.2%; p = 0.04). The expression of LDLR in normolipidemic subjects at baseline was twice as high compared to dyslipidemic subjects (p = 0.008). After twelve weeks of FO supplementation, no changes in LDLR expression were observed in normolipidemic subjects, while dyslipidemic subjects showed a significant down-regulation in LDLR expression (p = 0.02).

## Discussion

The effects of n-3 PUFAs on lipid levels in dyslipidemic conditions are well-known, however, only a few human studies investigated the underlying molecular mechanisms of gene expression levels in humans. Therefore, we performed a twelve-week lasting n-3 PUFA supplementation trial with normo- and dyslipidemic male subjects aiming to identify possible molecular pathways of n-3 PUFAs in humans.

Parts of this study have been published recently, presenting regulated pathways of the lipid metabolism after FO supplementation such as fatty acid biosynthesis, fatty acid elongation in mitochondria, fatty acid metabolism, glycerolipid metabolism, glycerophospholipid metabolism, arachidonic acid metabolism, linoleic acid metabolism and alpha-linoleic acid metabolism [25]. The present paper focuses on the influence of n-3 PUFAs on specific lipid metabolism associated gene expression.

Although the lipid metabolism is mainly located in the liver, several key regulators of the TG, HDL-C and LDL-C metabolism were found to be regulated on a transcriptional level in whole blood cells.

### TG metabolism

Supplementation of normo- and dyslipidemic subjects with n-3 PUFAs resulted in a decrease of TG levels by 23 and 28%, which is comparable to other studies [10]. Several genes which are likely to be involved in TG lowering were observed to be regulated (Figure 2). Consistent with findings from other studies [29,30], PPARα was up-regulated in dyslipidemic men after one week of n-3 PUFA supplementation. Transactivation of PPARs results in a repression of Apo CIII and Apo B, which, in turn, results in an enhanced lipoprotein lipase (LPL)-mediated catabolism of VLDL and reduced VLDL production [31]. Currently, this metabolic pathway is believed to be the main mechanism by which n-3 PUFAs reduce TG levels [10]. Expression changes of genes coding for Apo CIII and Apo B were not observed in this study, possibly because these factors are not expressed in blood cells but in other organs and tissues [32-34].

Two other transcription factors besides PPARα were regulated after n-3 PUFA supplementation. HNF6 and HNF1ß were down-regulated in dyslipidemic subjects at nearly all time-points. HNF6 is associated with several regulatory pathways influencing glucose metabolism, cholesterol metabolism, bile acid biosynthesis, as well as the synthesis and transport of serum carrier proteins [35]. Synergisms between HNF6 and HNF1α or between HNF1β and GATA6 could increase the activity of the HNF4α promoter [36], which, in turn, increases VLDL secretion. Since Apo CIII and Apo B are target genes of HNF4α [37], a reduced HNF4α expression, mediated by a down-regulation of HNF6 and HNF1ß, may reduce the VLDL secretion; the main TG-lowering effect of n-3 PUFAs. However, expression changes of HNF4α were not observed after FO supplementation in our experiments, most likely because HNF4α is expressed in liver, kidney, intestine, and pancreas [38]. Nevertheless, a robust suppression of the transcriptional HNF4α activity by n-3 PUFAs has been shown *in vitro*[39]. Furthermore, it was found that n-3 PUFAs reduce the expression of HNF4α in rat hepatocytes resulting in a decreased expression of Apo B and microsomal TG transfer protein, which suggests a reduced VLDL secretion [15]. Taken together, these findings suggest that this molecular pathway is a possible mechanism explaining the TG-lowering effect of n-3 PUFAs.

In addition to transcription factors, the expression of several other TG metabolism-associated genes was regulated after n-3 PUFA supplementation. Apo CII acts as a cofactor for LPL, which hydrolyses TGs in chylomicrons and VLDL particles to glycerine and free FAs. Therefore, constant Apo CII levels are crucial for LPL activation [40], ensuring efficient lipolysis of TG-rich lipoproteins. Studies have shown that dyslipidemic subjects without genetic disorders present higher Apo CII levels (7.0 mg/dl) than normolipidemic subjects (3.0 mg/dl) [41,42], which results in a disturbance of the Apo CII/LPL balance. Dyslipidemic subjects of our study consistently exhibited higher Apo CII mRNA expression levels than normolipidemic subjects at baseline. After FO treatment, Apo CII expression was down-regulated in dyslipidemic men suggesting a re-establishment of the Apo CII/LPL balance and an increased LPL-mediated TG clearance. In agreement, Zhang and co-workers [43] observed reduced plasma Apo CII levels in Chinese woman after increased fish intake (80 g fatty fish five times per week) over eight weeks.

Finally, the mRNA expression of three genes involved in TG synthesis, MOGAT3, MOGAT2 and DGAT1, were down-regulated in dyslipidemic men after n-3 PUFA supplementation. MOGATs and DGATs are key enzymes of the monoacylglycerol (MAG) pathway [44], which is primarily responsible for the re-synthesis of TGs from FAs and 2-MAG in enterocytes [45-47]. Besides the intestine, MOGAT2 and MOGAT3 are similarly expressed in the liver [44,47], whereas DGAT1 is expressed ubiquitously [48]. To the best of our knowledge, an effect of n-3 PUFAs on the expression of MOGAT2 and MOGAT3 has not yet been described. Knockdown of MOGAT3 by siRNA in liver-derived cells was followed by a reduced enzyme activity [44], which is accompanied by reduced intestinal dietary fat absorption in mice [49]. Due to their involvement in TG re-synthesis and secretion, it is believed that MOGAT2 and 3, but also DGAT1 are potential therapeutic drug targets for treating diet-induced dyslipidemia and obesity [44,49-51], whereas an activation of PPARα was suggested as the initial regulator [52]. We assume that the down-regulation of MOGAT2, MOGAT3 and DGAT1, observed in whole blood cells after FO supplementation, will similarly take place in enterocytes or hepatocytes, resulting in a reduced re-synthesis of TG and thus TG levels in circulation.

In addition, n-3 PUFA supplementation resulted in a marked reduction of Apo B48 levels (~30%), which might support this hypothesis. Apo B48 is an integral stabilizing component of re-synthesized TG containing chylomicrons [53]. Patients with hyperlipidemia and metabolic syndrome show elevated Apo B48 levels compared to normolipidemic subjects [54,55], which is connected with a higher cardiovascular risk. A reduced re-synthesis and secretion of TG in enterocytes would be accompanied with a reduced formation of chylomicrons and associated Apo B48. The lowering effect of n-3 PUFAs on levels of chylomicrons, their remnants, as well as Apo B48 has been shown in other studies [8]. However, beside this indirect effect, n-3 PUFAs might directly influence the synthesis of Apo B48.

### HDL cholesterol metabolism

**Figure 3:**
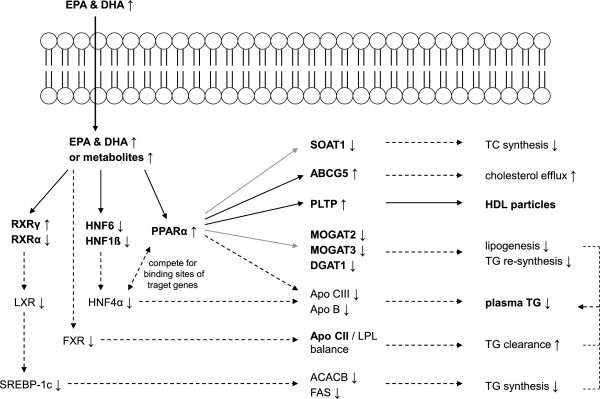
**Transcript levels of target genes in normolipidemic and dyslipidemic men.** Transcript levels of apolipoprotein CII (Apo CII) and low-density lipoprotein receptor (LDLR) was determined by qRT-PCR in normo- and dyslipidemic men before (t_0_) and after twelve weeks (t_12_) of fish oil supplementation. Pooled group samples were used in triplicates. Triplicates were averaged and corrected by two reference genes, glyceraldehyde-3-phosphate dehydrogenase (GAPDH) and ribosomal proteine S2 (RPS2). Corrected expressions were compared with baseline gene expression of normolipidemic subjects and relative expression changes are displayed.

As expected, FO supplementation increased HDL-C levels in both normo- and dyslipidemic subjects. The underlying HDL-C level raising mechanisms are not completely understood. Another target gene of PPARα that might be involved in raising HDL-C levels is PLTP [56-58], which was up-regulated in dyslipidemic subjects (Figure 3). PLTP can modulate HDL-C size and composition [59,60] by the transfer of phospholipids from TG-rich lipoproteins to HDL-C particles [61]. However, in contrast to our results, incubation of Hep G2 cells with EPA, DHA or arachidonic acid resulted in a decreased expression of PLTP [62]. Similarly, results from animal trials were inconsistent. Both PLTP deficiency and PLTP overexpression resulted in a significant reduction of HDL-C levels in the circulation [63].

### Total cholesterol and LDL cholesterol metabolism

A well-known effect of FO supplementation is a slight increase of LDL-C levels after n-3 PUFA supplementation [5], which is most likely the result of an increased conversion of VLDL-C to LDL-C [13]. Although we observed a considerable increase in LDL-C levels in dyslipidemic subjects, the expression of several regulated genes in the same subjects indicate not only cholesterol-lowering, but also an enhancing effect (Figure 3). LDLR, which is expressed in nearly all cells, but predominantly in liver cells, transports cholesterol-rich lipoprotein particles, preferably LDL-C, via endocytosis into the cell, resulting in LDL-C clearance [64]. Even though high cholesterol concentrations inhibit the LDLR expression [65], LDLR mRNA levels in dyslipidemic subjects were two times higher compared to normolipidemic subjects. After FO supplementation, the LDLR expression was down-regulated in dyslipidemic subjects, which is in agreement with other studies [66]. The reduced LDLR expression suggests a diminished LDL-C clearance and could be another mechanism explaining the n-3 PUFA-induced rise in LDL-C levels.

In contrast, the regulation of two other factors points to a cholesterol-lowering effect. The half-transporters ABCG5 and ABCG8 play a pivotal role in the regulation of dietary cholesterol transport into the intestinal and biliary lumen for faecal excretion [67]. The up-regulation of ABCG5 in dyslipidemic subjects might induce a pathway that results in increased cholesterol efflux. In agreement with our results, two animal studies revealed an increased hepatic ABCG5 and ABCG8 expression in mice after FO feeding [68,69]. The regulation might be triggered by PPARA, since PPARα agonists caused an up-regulation of ABCG5 expression in hypercholesterolemic subjects [70].

Similarly, the down-regulation of SOAT1, observed in dyslipidemic subjects after FO supplementation, suggests a cholesterol-lowering effect. SOATs play important roles in cellular cholesterol homeostasis in various tissues [71]. Accordingly, a down-regulation of SOAT1 expression after n-3 PUFA treatment has been observed in human breast cancer cells [72].

### Limitations

The study has a number of potential limitations. As already mentioned, it is critical to investigate the regulative effect of n-3 PUFAs on the expression of lipid metabolism-associated genes in whole blood cells instead of liver cells. However, the sampling of biopsies from the liver in this study was precluded for ethical and medical reasons. We similarly abstained from isolating lymphocytes or peripheral blood mononuclear cells (PBMCs), an approach which was chosen by two other groups investigating a similar question [73,74], since cell fractioning steps involve stress-induced alterations in gene expression profiles [75]. However, many lipid metabolism-associated genes are also expressed in nucleated blood cells: For example, PPARα is expressed in monocytes, macrophages and lymphocytes [76,77], while HNF6 and DGAT were found to be expressed in PBMCs [48,78]. In addition, the pooling of RNA samples reduces inter-individual variation, enabling one to focus on the effects of FO supplementation on the population level in contrast to an individual level [79]. However, the approach of sample pooling provides several limitations, primarily the reduction of statistical power.

## Conclusion

This pilot study suggests molecular pathways on how n-3 PUFAs affect lipid metabolism. Although the study is limited by the usage of whole blood cells obviating strong conclusions about lipid metabolism, several lipid metabolism-associated genes were shown to be regulated on a transcriptional level in dyslipidemic subjects, including transcription factors PPARα, RXRα, RXRγ, HNF6, and HNF1ß, as well as other lipid regulators, MOGAT2, MOGAT3, DGAT1, Apo CII, PLTP, LDLR, ABCG5, and SOAT1. We assume that this transcriptional regulation will equally take place in cells of the liver or other tissues. Given this assumption, n-3 PUFAs activate several transcription factors resulting in the regulation of numerous target genes which, in turn, affect multiple lipid regulators. Accordingly, the results give indications for (1) decreased TG levels as a result of an enhanced VLDL catabolism and reduced VLDL production, as well as a decreased TG re-synthesis in enterocytes and hepatocytes, while (2) increased HDL-C levels may be the result of an increased transfer of phospholipids from TG-rich lipoproteins to HDL-C particles. Finally, (3) LDL-C levels may be influenced in both directions: A decreased LDL-C clearance may result in rising LDL-C levels, on the one hand, while an increased cholesterol efflux and a reduced cholesteryl ester synthesis result in decreasing LDL-C levels, on the other hand. Future studies combining gene expression, metabolic markers and clinical end points need to clarify the significance of the hypothesized molecular mechanisms.

## Abbreviations

ABCG5: ATP-binding cassette sub-family G member 5; Apo: Apolipoprotein; DGAT: Diacylglycerol O-acyltransferase; DHA: Docosahexaenoic acid; EPA: Eicosapentaenoic acid; FA: Fatty acid; FO: Fish oil; GAPDH: Glyceraldehyde-3-phosphate dehydrogenase; GCP: Good clinical practice; HDL-C: High-density lipoprotein cholesterol; HNF: Hepatic nuclear factor; LDL-C: Low-density lipoprotein cholesterol; LDLR: LDL receptor; LPL: Lipoprotein lipase; MOGAT: 2-acylglycerol O-acyltransferase; n-3 PUFA: Omega-3 polyunsaturated fatty acid; PPAR: Peroxisome proliferator-activated receptor; PBMC: Peripheral blood mononuclear cells; PLTP: Phospholipid transfer protein; qRT-PCR: Quantitative real-time polymerase chain reaction; RPS2: Ribosomal protein S2; RXR: Retinoid X receptor; SOAT1: Sterol O-acyltransferase 1; SREBP: Sterol regulatory element-binding protein; t: Time point; TC: Total cholesterol; TG: Triacylglycerol; VLDL: Very low-density lipoprotein.

## Misc

Andreas Hahn and Jan Philipp Schuchardt contributed equally to this study.

## Competing interests

The authors declare that they have no competing interests.

## Authors’ contributions

All authors have read and approved the final manuscript. SS was involved in the study, experimental design, data analysis, interpretation, and manuscript writing. The study was mainly performed by SS. FS was involved in the experimental design and informed advice. KOM was involved in the experimental design, data analysis and manuscript editing. JPS was involved in study design, data interpretation and manuscript writing. JW was involved in manuscript writing. The group leader of the Institute of Technical Chemistry, TS, was involved in the study design and manuscript editing. The group leader of the Institute of Food Science and Human Nutrition, AH, was involved in the study design and manuscript editing. Both JPS and AH were coordinators of the study.
